# Effect of fluid loading during hypovolaemic shock on caspofungin pharmacokinetic parameters in pig

**DOI:** 10.1186/cc10455

**Published:** 2011-09-20

**Authors:** Antoine Roch, Christian Woloch, Dorothée Blayac, Caroline Solas, Sylvie Quaranta, Vincent Mardelle, Matthias Castanier, Laurent Papazian, Emmanuelle Sampol-Manos

**Affiliations:** 1 Aix-Marseille Univ, URMITE CNRS-UMR 6236, 27 boulevard Jean Moulin, 13005 Marseille, France; 2 Laboratoire de Pharmacocinétique et de Toxicologie, Hôpital de la Timone, 264 rue Saint Pierre, 13005, Marseille, France; 3 Institut de médecine tropicale du service de santé des armées, 58 boulevard Charles Livon, 13007, Marseille, France; 4 APHM, Hôpital Nord, Réanimation, Chemin des Bourrely 13915, Marseille, France

**Keywords:** echinocandin, pharmacokinetics, intensive care unit, lung

## Abstract

**Introduction:**

Caspofungin treatment is frequently initiated in shock patients. In the present study, we investigated the influence of hypovolaemic shock requiring fluid loading on the plasma and pulmonary pharmacokinetic parameters of caspofungin in the pig.

**Methods:**

After being anaesthetised and mechanically ventilated, 12 pigs were bled to induce a two-hour deep shock and resuscitated using normal saline based on haemodynamic goals. A one-hour infusion of 70 mg of caspofungin was started at the beginning of the resuscitation period. The lungs were removed four hours after caspofungin administration. Sixteen animals served as controls without haemorrhage. Caspofungin concentrations were measured by using high-performance liquid chromatography, and a two-compartment population pharmacokinetic analysis was performed.

**Results:**

In the shock group, the volume of blood removed was 39 ± 7 mL/kg and a volume of 90 ± 17 mL/kg saline was infused throughout the resuscitation period. The extravascular lung water index was higher in the shock group (9.3 ± 1.6 mL/kg vs 5.7 ± 1 mL/kg in the control group; *P *< 0.01). In the shock group, the median (interquartile range) maximal plasma concentration was 37% lower than in the control group (21.6 μg/mL (20.7 to 22.3) vs 33.1 μg/mL (28.1 to 38.3); *P *< 0.01). The median area under curve (AUC) from zero to four hours was 25% lower in the shock group than in the control group (60.3 hours × μg/mL (58.4 to 66.4) vs 80.8 hours × μg/mL (78.3 to 96.9); *P *< 0.01), as was the median lung caspofungin concentration (1.22 μg/g (0.89 to 1.46) vs 1.64 μg/g (1.22 to 2.01); *P *< 0.01). However, the plasma-to-tissue ratios were not different between the groups, indicating that lung diffusion of caspofungin was not affected after shock followed by fluid loading. Pharmacokinetic analysis showed that the peripheral volume of distribution of caspofungin and intercompartmental clearance were significantly higher in the shock group, as was the total apparent volume of distribution.

**Conclusions:**

Hypovolaemic shock followed by fluid loading in the pig results in a significant increase in the apparent volume of distribution of caspofungin and in a decrease in its plasma and pulmonary exposition. Although our model was associated with capillary leakage and pulmonary oedema, our results should be generalised to the septic shock with caution. Future investigations should focus on monitoring plasma caspofungin concentrations and optimal caspofungin dosing in shock patients.

## Introduction

Fungal infections are an important cause of mortality in ICU patients [1]. Moreover, the prevalence of *Candida *infections has increased during the past decade or so [2], and recent data indicate that invasive pulmonary aspergillosis may be an underestimated opportunistic fungal infection in ICU patients [3].

Drug pharmacokinetic parameters in ICU patients are often different from those in healthy patients, notably because of physiological alterations induced by sepsis [4-6]. Besides changes in tissue perfusion, protein binding and clearance, the observed alterations in membrane permeability and the subsequently required fluid loading increase the volume of distribution of many drugs, which results in insufficient dosages of some antibiotics in a significant proportion of ICU patients [6-8].

Echinocandins such as caspofungin are semisynthetic lipopeptides with a pathogen-specific mechanism for noncompetitive inhibition of biosynthesis of the fungus cell wall enzyme complex 1,3-β-D-glucan [9,10]. Echinocandins have documented *in vivo *activity against *Candida *spp. [11] and *Aspergillus *spp. [12]. They are recognised as antifungal agents of choice in ICU patients [13]. The antifungal activity of echinocandins is concentration- and time-dependent and the ratio of area under the curve (AUC) to the minimal inhibitory concentration has been shown to be a good descriptor of their exposure-response relationship [14-16]. Therefore, the adequacy of the initial dose is an important point in the efficacy of echinocandins [17]. In the ICU, caspofungin treatment is frequently initiated in patients who present with septic shock, which is notably characterised by hypovolaemia requiring fluid loading for several hours. The exposition and distribution of caspofungin may be affected in these patients, resulting in insufficient plasma and tissue concentrations [18].

The selective study of the impact of hypovolaemic shock and fluid loading is difficult in ICU patients, who have many comorbidities that can affect caspofungin pharmacokinetics, such as renal insufficiency, need for dialysis, widely variable albumin levels and concomitant medications. For this reason, in the present study, we specifically investigated the influence of hypovolaemic shock followed by fluid loading on the plasma pharmacokinetic parameters and the pulmonary diffusion of caspofungin in a clinically relevant pig model. Our protocol was designed to mimic, with good reproducibility, deep hypovolaemic shock followed by resuscitation concomitantly with caspofungin administration.

## Materials and methods

The study protocol was approved by the ethics committee of the Institut de médecine tropicale du service de santé des armées, Marseille, France. Twenty-eight four-month-old pigs weighing 40 ± 3 kg (mean ± SD) were studied.

### Anaesthesia and monitoring

Since inducing deep, sustained shock is inconsistent with spontaneous ventilation, the pigs were anaesthetised and mechanically ventilated. After premedication by intramuscular injection of 2 mg/kg midazolam, anaesthesia was induced and maintained by an intravenous infusion of midazolam (0.2 mg/kg/hour), fentanyl (0.01 mg/kg/hour) and pancuronium (0.3 mg/kg/hour). A normal saline solution (3 mL/kg/hour) was infused continuously throughout experiment. The animals were tracheotomised and ventilated with a tidal volume of 10 mL/kg and a fraction of inspired oxygen of 0.5 (Servo 900C ventilator; Siemens-Elema AB, Solna, Sweden). The respiratory rate was adjusted to obtain an arterial partial pressure of carbon dioxide between 35 and 45 mmHg. A 5-French catheter (Edwards Lifesciences, Irvine, CA, USA) was inserted into the left internal jugular vein for fluid loading, and another 5-French catheter was inserted into the left carotid artery for blood withdrawal and monitoring of mean arterial pressure (MAP). A pulmonary artery catheter (Edwards Lifesciences) was inserted through the right external jugular vein into the right pulmonary artery for measurement of cardiac index (CI). Identification of the CI end point during the haemorrhage and resuscitation periods was assessed using the Vigilance Continuous Cardiac Output Monitor (Baxter, Deerfield, IL, USA). CI values recorded for analysis were obtained after injection of three 12-mL boluses of 5% glucose solution between 0°C and 5°C via a closed system at end inspiration. Mixed venous oxygen saturation (SVO_2_) in pulmonary arterial blood was measured using a CO-oximeter (278 Blood Gas System; Ciba Corning, Medfield, MA, USA).

### Experimental groups

**Figure 1:**
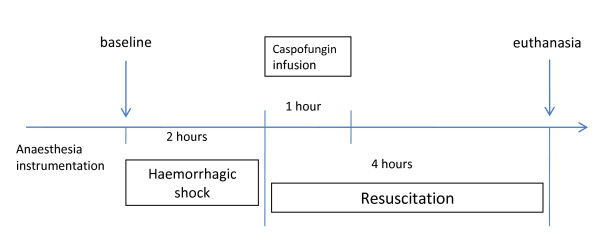
**Study protocol in the shock group**.

In the shock group (*n *= 12), the animals were bled through the carotid catheter (Figure 1). Blood withdrawal was started at a rate of 0.8 to 1 mL/kg/minute and continued until all three of the following end points were achieved: MAP of 40 to 45 mmHg, decrease in CI greater than 40% and SVO_2 _lower than 30%. End points were maintained for a period of 120 minutes by means of further bleeding. During the four-hour resuscitation period, the animals received isotonic saline at a rate of 2 mL/kg/minute. The infusions were stopped when the CI reached 90% of the baseline value and SVO_2 _was above 50%, and the infusions were then adjusted to keep these parameters constant until the animals were killed. At the beginning of the resuscitation period, the animals received a one-hour intravenous infusion of 70 mg of caspofungin acetate (Cancidas; Merck Sharp & Dohme-Chibret, Paris, France) diluted in 50 mL of saline, which was injected into the right atrium through the proximal lumen of the pulmonary arterial catheter via a pump-driven syringe. A second group of animals (*n *= 16) used as controls was anaesthetised and ventilated, but they were not subjected to bleeding and fluid loading. These animals were also killed four hours after the start of the caspofungin infusion.

### Caspofungin sampling and assay

EDTA-blood samples were drawn through a dedicated arterial catheter just before and 15, 30, 60 and 90 minutes and 2 hours, 3 hours and 4 hours after the caspofungin infusions were started in both groups. Then the animals were immediately killed, and lung tissue samples (2 cm × 2 cm × 2 cm) were surgically removed from nondependent parts of both the lower and upper lobes after sternotomy. Plasma was obtained by blood centrifugation at 1,500 *g *for 15 minutes. Plasma and lung samples were immediately stored at -80°C. The remaining lung tissues were used for the determination of extravascular lung water indexed to body weight (EVLWI) by using a gravimetric method as previously described [19]. Caspofungin acetate was kindly provided by Merck Sharp & Dohme-Chibret. Plasma and lung caspofungin concentrations were determined by using a validated high-performance liquid chromatography method with fluorescence detection [20] after a solid-phase extraction (Bond Elut™ column; Agilent Technologies, Santa Clara, CA, USA). Caspofungin and the internal standard (IS) propranolol were separated on a LiChrospher column (100 RP-8e (5 μm); Merck Chemicals KGaA, Darmstadt, Germany) using a mobile phase consisting of acetonitrile and 0.1% trifluoroacetic acid buffer (200:300 vol/vol) at a flow rate of 1 mL/minute. The retention times were 6 and 22 minutes for IS and caspofungin, respectively. The linearity range was 0.3 to 7.5 mg/L (*r*^2 ^> 0.998). The lower limit of quantification, defined as the lowest concentration that could be determined with a relative error and precision (relative standard deviation) less than 20%, was 0.3 μg/mL. Intraday and interday levels of interassay precision were 1.91% and 4.84% (0.5 μg/mL), 6.94% and 10.4% (3 μg/mL) and 7.84% and 12% (5 μg/mL) for serum samples. The maximal concentration (*C*_max_) was measured at the end of the infusion.

Lung samples were weighed (200 ± 150 mg), homogenised with a 1.5-mL saline solution, ground for 15 minutes and centrifuged twice at 10,000 rpm for 10 minutes. The supernatant liquid was separated, and then the IS solution was added to 1 mL of supernatant liquid. For calibration, we spiked different caspofungin and IS concentrations in saline (matrix of pretreatment). Lung caspofungin concentrations (*C*_t_) were determined four hours after starting the caspofungin infusion and are expressed as micrograms per gram of lung tissue. The tissue-to-plasma ratio for each group was calculated as *R *= (*C*_t_/*C*_p_) × 100, where *C*_p _is the caspofungin plasma concentration at four hours.

### Caspofungin pharmacokinetic analysis

The AUC from zero to four hours (AUC_0→4 hours_) was determined using the trapezoidal rule. The concentration vs time data for both groups were modelled using the population approach method implemented in Monolix version 3.1 release 2 software. A two-compartment mammillary model was developed and parameterised in terms of central volume of distribution of caspofungin (*V*_1_) and elimination clearance of caspofungin (CL), peripheral volume of distribution of caspofungin (*V*_2_) and intercompartmental clearance of caspofungin (Q). The total apparent volume of distribution of caspofungin (*V*_d_) was calculated as *V*_1 _+ *V*_2_. The interindividual variability (ω) for each parameter and the residual variability of the model (σ) were estimated.

### Statistical analysis

Statistical calculations were performed using the SPSS 15.0 software package (SPSS Inc., Chicago, IL, USA). The results are expressed as means ± standard deviations (SD) if data were normally distributed or as medians (interquartile ranges). Two-way analysis of variance for repeated measures was used to analyse the effect of group and time on MAP. Tukey's test was used for *post hoc *analyses. The Mann-Whitney rank-sum test was used to compare plasma caspofungin concentrations, AUC_0→4 hours_, lung caspofungin concentrations (*C*_t_), plasma-to-tissue ratios and caspofungin population pharmacokinetic parameters between groups. *P *≤ 0.05 was considered statistically significant.

## Results

### Haemodynamics and lung water

**Figure 2:**
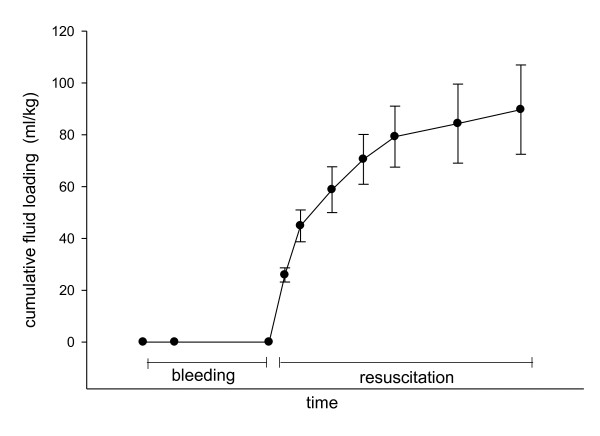
**Time evolution of cumulative fluid loading in the shock group**. The results are expressed as means ± standard deviation (SD).

**Figure 3:**
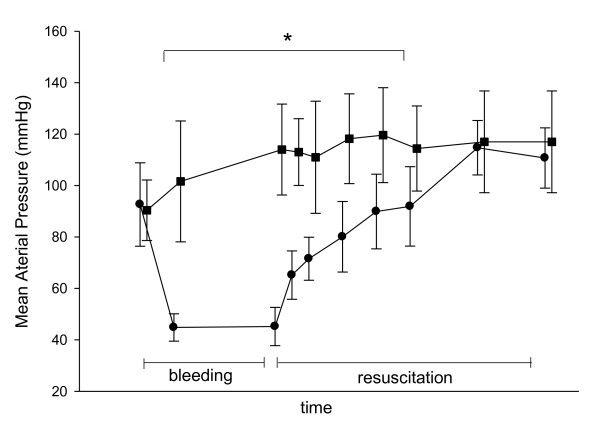
**Mean arterial pressure in the control group (squares) and shock group (circles) at baseline and after 30 minutes and 120 minutes of bleeding, as well as after 15, 30, 60, 90 and 120 minutes of resuscitation and after 3 and 4 hours of resuscitation**. The results are expressed as means ± standard deviation (SD). *P < 0.001 between groups (Tukey's test).

In the shock group, the volume of blood removed was 39 ± 7 mL/kg, and a volume of 90 ± 17 mL/kg of isotonic saline was infused throughout the resuscitation period (Figure 2). MAP was progressively restored during fluid loading and was not different from the control group from three hours of resuscitation (Figure 3). EVLWI was higher in the shock group than in the control group (9.3 ± 1.6 mL/kg vs 5.7 ± 1 mL/kg; *P *< 0.01).

### Caspofungin plasma pharmacokinetics

**Figure 4:**
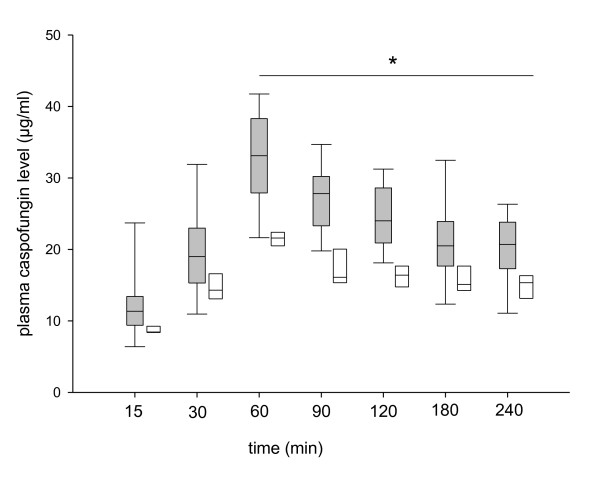
**Caspofungin concentration time profile in the shock group (white plots) and in the control group (grey plots)**. Each plot represents the median and 25th and 75th percentile values. Bars represent the 5th and 95th percentiles. *P < 0.01 vs control group.

The caspofungin plasma concentration profile over a four-hour period in animals in the shock and control groups is presented in Figure 4. Median *C*_max _was 35% lower in the shock group (*P *< 0.002 vs control group) (Figure 4 and Table 1), and the caspofungin concentration remained significantly lower in the shock group until four hours after caspofungin infusion was started, resulting in a 25% lower median AUC_0→4 hours _(Table 1). Whereas *V*_1 _and CL from the central compartment were not different between groups, Q, *V*_2 _and *V*_d _were higher in the shock group (Table 2).

**Table 1:** Caspofungin pharmacokinetic parameters in plasma and lung tissue

	Shock group (*n *= 12)		Control group (*n *= 16)		
Caspofungin parameters	Median	IQR	Median	IQR	*P *value
Plasma					
*C*_max _(μg/mL)	21.6	20.7 to 22.3	33.1	28.1 to 38.3	0.002
AUC_0→4 hours _(μg/hour/mL)	60.3	58.4 to 66.4	80.8	78.3 to 96.9	0.009
*C*_p _(μg/mL)	16.1	12.5 to 17.4	20.7	17.4 to 23.1	0.007
Lung tissue					
*C*_t _(μg/g)	1.22	0.89 to 1.46	1.64	1.22 to 2.01	0.001
Ratio (%)	7.76	6.41 to 9.42	7.28	6.2 to 9.5	0.862

AUC_0→4 hours_: area under the curve of caspofungin plasma concentration from zero to four hours; *C*_max_: maximum caspofungin concentration; *C*_p_: caspofungin plasma concentration at four hours; *C*_t_: lung caspofungin concentration at four hours; IQR: interquartile range. Ratio = (*C*_t_/*C*_p_) × 100; *P *values are for shock group vs control group.

**Table 2:** Caspofungin population pharmacokinetic parameters in plasma

	Shock group (*n *= 12)		Control group (*n *= 16)	
Caspofungin parameters	Estimate	RSE (%)	Estimate	RSE (%)
CL (L/hour)	0.21	26	0.29	20
*V*_1 _(L)	0.96	45	0.98	24
Q (L/hour)	8.16*	39	4.23	30
*V*_2 _(L)	2.8*	15	1.43	12
Vd (L)	3.9*	-	2.5	-
ω (CL) (%)	25	21	75	39
ω (*V*_1_) (%)	10	37	80	36
ω (Q) (%)	10	32	54	35
ω (*V*_2_) (%)	10	35	19	34
σ (%)	28	13	34	8

CL: central elimination clearance; Q: intercompartmental clearance; RSE: relative standard error;

*V*_1_: central volume of distribution; *V*_2_: peripheral volume of distribution; *V*_d_: total apparent volume of distribution; σ: residual variability of the model; ω: interindividual variability. *P < 0.05 vs control group.

### Lung caspofungin concentrations

Caspofungin concentrations were not significantly different between the lower and upper lung lobes or between the left and right lungs. Therefore, values from the four sampled lobes were averaged, and the mean lung concentration is reported. We observed a 25% lower median lung caspofungin concentration in the shock group than in the control group (Table 1). However, no significant difference was observed between plasma-to-tissue ratios in the two groups, indicating that lung diffusion of caspofungin was not affected after shock followed by fluid loading.

## Discussion

In ICU patients, septic shock is common and carries a high mortality rate [21]. In this situation, both the precocity and adequacy of antimicrobial therapy are major prognostic factors [22]. The antifungal activity of caspofungin has been shown to be concentration-dependent *in vitro *and *in vivo *[14,15]. Moreover, the adequacy of the initial dose may be an important point in the efficacy of echinocandins [17]. Therefore, it is critical to administer a sufficient dose, especially in the first days of treatment. Our results show that hypovolaemic shock followed by fluid loading in the pig results in a significant decrease in early plasma caspofungin exposition, as reflected by a lower maximal concentration and a lower AUC_0→4 hours_. Fluid loading was associated with a decrease in the pulmonary concentration of caspofungin.

In the early clinical development program for caspofungin, a target concentration of 1 μg/mL was used as a guide for concentrations likely to be efficacious. This target was determined on the basis of *in vitro *susceptibility test results and was selected as the concentration that exceeded the minimal inhibitory concentration at which 90% of *Candida *spp. isolates were inhibited [23]. In healthy humans, a single dose of 70 mg resulted in a mean plasma *C*_max _of 12.04 μg/mL (95% confidence interval, 10.87 to 13.33) and a concentration at 24 hours (trough concentration) of 1.42 μg/mL (95% confidence interval, 1.18 to 1.71) with linear plasma pharmacokinetics [24]. On the basis of these studies, a once-daily dosing regimen of 50 mg was considered adequate for efficacy in the treatment of fungal infections. However, a loading dose of 70 mg is recommended, since trough caspofungin plasma concentrations fell below the target concentration of 1 μg/mL for the first two days of administration in the absence of a loading dose.

The pharmacokinetics of caspofungin have been recently studied in a cohort of surgical ICU patients [18]. In that study, caspofungin exhibited wide variations in 24-hour trough plasma concentrations during the whole treatment period. Although the mean trough concentration at day 2 of treatment was slightly higher than reported in healthy humans [21] and exceeded 1.0 μg/mL in 75% of patients, it failed to reach this target concentration in 25% of them [18]. Therefore, the authors concluded that a dose adjustment may be necessary in some ICU patients, based on the monitoring of plasma concentrations. Although 29 of the 40 included patients received vasopressors for shock, neither the need for fluid loading nor its impact on caspofungin pharmacokinetics was reported [18]. The authors attributed the variability of caspofungin pharmacokinetics not only to the effect of supportive therapies such as fluid loading, haemodialysis and catecholamines but also to patient-specific parameters such as albuminaemia, organ dysfunction and weight. In the present study, we specifically evaluated the effect of hypovolaemic shock requiring large fluid loading with a positive fluid balance exceeding 50 mL/kg. Our observed decrease in plasma and pulmonary caspofungin exposition in this situation points out that this factor may largely contribute to the underdosing reported in ICU patients [18]. Since the volume of distribution of echinocandins is likely to correspond to the extracellular volume [25], the massive fluid loading required to restore and maintain haemodynamics observed in the present study or in shock patients in general is likely to be responsible for the increase in *V*_2 _and *V*_d _as well as in the subsequent decrease in caspofungin's early plasma and tissue concentrations. Whether a subsequent modification of CL would affect its pharmacokinetics in this situation requires further studies. Notably, the haemodynamic failure observed while initiating treatments such as caspofungin in septic patients is frequently even more marked than in our present study [26]. Therefore, it is possible that caspofungin concentrations during the first days of treatment may be insufficient in a significant number of ICU patients with marked haemodynamic instability [18,27].

With regard to lung tissue concentrations, our observed decrease in plasma concentration had a direct impact on pulmonary concentrations measured four hours after administration. These data signify that insufficient initial plasma concentrations may result in lowered concentrations at the site of the infection. As is true during sepsis, our model was characterised by lung oedema and inflammation [19], which did not affect the pulmonary diffusion of caspofungin or influence its distribution to the different lung lobes.

Our study has several limitations. First, we used a haemorrhagic shock model rather than an invasive fungal infection model. Animal models of invasive candidiasis or aspergillosis [28] are mainly used to study the efficacy of antifungal therapies. However, these models do not induce hypovolaemic shock such as required for the present study. A commonly used model of sepsis is lipopolysaccharide (LPS) injection [29], which reproduces bacterial sepsis. This model may be more representative than haemorrhagic shock of the capillary leakage observed during human sepsis and could have different influences on the distribution of caspofungin. However, this model has unpredictable effects on pharmacokinetics, since haemodynamic effects are very variable and pathophysiological changes caused by LPS administration can potentially affect all aspects of drug metabolism [29]. Moreover, our model is also characterised by capillary leakage and vascular failure, as attested by requirements for fluid loading and the observed increase in lung water. Second, we did not study the long-term pharmacokinetics of caspofungin, since our model is inconsistent with a prolonged survival of the animals without other therapeutic interventions. Considering the pharmacokinetic linearity of caspofungin [24], the decrease in caspofungin concentrations that we observed is likely to affect trough plasma and tissue concentrations. However, further studies are required to clarify whether tissue concentrations may be lowered during the course of several days. Indeed, therapeutic concentrations of caspofungin have been shown to persist at the site of infection well after serum concentrations fall below the minimal inhibitory concentration, and the terminal half-life of this drug has been suggested to be heavily influenced by the accumulation of caspofungin in tissues [28]. Third, we did not study which dose of caspofungin would result in similar exposition in shock and control animals. Recently, the use of higher caspofungin doses has been shown to be safe in healthy adults and could be evaluated in ICU patients [30]. Finally, we did not measure caspofungin concentrations in other tissues. However, lung concentrations of caspofungin are representative of those measured in various organs [31].

## Conclusions

The present study shows that hypovolaemic shock requiring a massive fluid loading is responsible for a marked decrease in plasma and pulmonary caspofungin early exposition. Although our results are insufficient to recommend higher caspofungin doses in septic shock patients, they warrant for clinical studies to define the optimal dosing of caspofungin at the initial stage of fungal sepsis and to assess the usefulness of its concentration monitoring.

## Key messages

• Hypovolaemic shock requiring massive fluid loading is responsible for a marked decrease in plasma and pulmonary caspofungin early exposition.

• Lung diffusion of caspofungin was not affected in these conditions.

• Investigators in future studies should study the optimal dosing of caspofungin at the initial stage of fungal sepsis and the usefulness of monitoring its concentration.

## Abbreviations

σ: residual variability of the model; ω: interindividual variability; AUC_0→4 hours_: area under the curve of caspofungin plasma concentration from zero to four hours; CI: cardiac index; CL: elimination clearance of caspofungin; *C*_max_: maximal plasma concentration of caspofungin; *C*_p_: caspofungin plasma concentration at four hours; *C*_t_: lung caspofungin concentration at four hours; EVLWI: extravascular lung water indexed to body weight; MAP: mean arterial pressure; Q: intercompartmental clearance of caspofungin; SVO_2_: mixed venous oxygen saturation; *V*_1_: central volume of distribution of caspofungin; *V*_2_: peripheral volume of distribution of caspofungin; *V*_d_: total apparent volume of distribution of caspofungin.

## Competing interests

The authors declare that they have no competing interests.

## Authors' contributions

AR and ES conceived the study, participated in coordination and drafted the manuscript. CW, DB, VM and MC collected data and helped to draft the manuscript. CS and SQ participated in coordination and drafted manuscript. LP participated in the design of the study and helped to draft the manuscript. All authors read and approved the final manuscript.
